# Revisiting the Effects of Gender Diversity in Small Groups on Divergent Thinking: A Large-Scale Study Using Synchronous Electronic Brainstorming

**DOI:** 10.3389/fpsyg.2021.723235

**Published:** 2021-10-11

**Authors:** Laurine Peter, Nicolas Michinov, Maud Besançon, Estelle Michinov, Jacques Juhel, Genavee Brown, Eric Jamet, Anthony Cherbonnier, ProFAN Consortium

**Affiliations:** Rudmann Ocyna (Social Psychology Laboratory, University of Lausanne, Lausanne, Switzerland); Ofosu Nana (Faculty of Psychology and Educational Sciences, University of Geneva, Geneva, Switzerland); (Humanities Department, University of Ferrara, Ferrara, Italy); (Pact Lab, Nothumbria University, Newcastle upon Tyne, United Kingdom); (Cognitive Psychology Laboratory, Aix-Marseille Univ, Marseille, France); (LaRAC, University of Grenoble Alpes, Grenoble, France); (Laboratory of Psychology: Cognition, Behavior, Communication, Univ Rennes, Rennes, France); (Social and Cognitive Psychology Laboratory, University of Clermont Auvergne, Clermont-Ferrand, France); (LIMOS, University of Clermont Auvergne, Clermont-Ferrand, France); ^1^Laboratory of Psychology: Cognition, Behavior and Communication (LP3C), Department of Psychology, Univ Rennes, Rennes, France; ^2^Pact Lab, Department of Psychology, Northumbria University, Newcastle upon Tyne, United Kingdom

**Keywords:** gender diversity, electronic brainstorming, collaborative creativity, idea generation, divergent thinking, “solo” groups

## Abstract

Numerous studies have examined the effects of gender diversity in groups on creative performance, and no clear effect has been identified. Findings depend on situational cues making gender diversity more or less salient in groups. A large-scale study on two cohorts (*N* = 2,261) was conducted among business students to examine the impact of the gender diversity in small groups on divergent thinking in an idea-generation task performed by synchronous electronic brainstorming. Participants were automatically randomized in three- or four-member groups to generate ideas during 10 min on a gendered or neutral task. Then, five categories of groups where the proportion of men/women in groups varied from three/four men to three/four women were compared to examine creative performance on three divergent thinking measures (fluency, flexibility, and originality). A Multivariate Generalized Linear Mixed Model (mGLMM) showed greater fluency in all-women groups than in other groups (except mixed-gender groups composed of two men and two women), and more specifically “solo” groups composed of a single woman/man among a majority of men/women. For flexibility and originality, the superiority of all-women groups was found only in comparison to “solo” groups composed of a single woman. As gender differences are more salient in “solo” groups than in other groups *faultlines* may appear in groups, leading to a deleterious impact on creative performance.

## Introduction

With the increasing proportion of women in the workforce and (virtual) teams in organizations, group diversity, and more specifically gender diversity, has emerged as a growing research interest in psychological research (Bradley et al., 2021). Beyond its impact on team performance, it is reasonable to suppose that gender diversity may also have an impact on creative performance. In the creativity literature, two types of creative thinking are generally distinguished (Guilford, 1950): Divergent thinking is used to find as many ideas as possible while convergent thinking allows the combination and association of several ideas or stimuli leading to a single production or solution (Cropley, 2006; Runco and Acar, 2012; Runco and Jaeger, 2012). In the present study, as in plethora of studies in social psychology which examine performance on creative tasks, creativity will be measured by a classic divergent thinking task, i.e., brainstorming (see for reviews, Paulus, 2000; Paulus and Nijstad, 2003; Paulus et al., 2012). As divergent thinking is considered an excellent indicator of creativity (Runco and Acar, 2012), a number of studies have examined divergent thinking using the (electronic) brainstorming technique asking participants to generate as many ideas as possible (Woolley et al., 2010; Engel et al., 2014; Malone and Bernstein, 2015; Coursey et al., 2020; see Maaravi et al., 2020 for a review).

Diversity in groups is one variable which may play a role in creativity in groups and teams. Diversity is determined by the process by which group members are different from one another on one or several criteria, and refers to “any attribute that another person may use to detect individual difference” (Williams and O’Reilly, 1998, p. 81). It has been demonstrated that group and team diversity may have an impact on performance (Rogelberg and Rumery, 1996; van Knippenberg et al., 2004; Horwitz, 2005; Myaskovsky et al., 2005; Horwitz and Horwitz, 2007; van Knippenberg and Schippers, 2007; Salazar et al., 2017; Moreland et al., 2018; van Knippenberg and Mell, 2020), and notably on creative performance (Milliken et al., 2003; Kurtzberg, 2005; Shin et al., 2012; Paulus and van der Zee, 2015; Abraham, 2016; Paulus et al., 2016; Coursey et al., 2020; Hoever and van Knippenberg, 2021). Among the different attributes of diversity in groups, gender diversity has been studied and findings have been mixed (Cady and Valentine, 1999; Baer and Kaufman, 2008; Baer et al., 2008; Díaz-García et al., 2013; Pluut and Curşeu, 2013). It is proposed in the present research that mixed findings observed in the literature may depend, at least in part, on saliency of gender differences in groups. In other words, the impact of gender diversity in groups partly depends on the possibility for individuals to divide groups or teams into subgroups on the basis of situational cues rendering gender more or less salient, such as stereotypical task content or saliency of gender characteristics within groups (Pearsall et al., 2008; Jehn and Bezrukova, 2010; Bell et al., 2011; van Dijk et al., 2012). When division occurs in groups, negative effects of gender diversity can be observed, and they may be accentuated when the task is gender specific, for example, when group members have to design a men’s electric razor (Pearsall et al., 2008). In the current study we use two idea-generation tasks (one stereotypically masculine and neutral) with a large sample of same and mixed gender groups to examine the influence of gender diversity on creative task performance.

### Group Diversity and Creative Performance

Intuitively, people tend to think that the diversity in groups enhances creativity, and consistent with this intuition, empirical research has demonstrated that individuals in heterogeneous groups produce more creative ideas than those in homogenous groups (Schruijer and Mostert, 1997; Curşeu et al., 2007). However, reviews of the literature suggested that the relationship between group diversity and creativity is more complex, and it strongly depends on the type of diversity taken into consideration (van Knippenberg and Schippers, 2007; Hülsheger et al., 2009; Pluut and Curşeu, 2013; Paulus et al., 2016, 2018; Yakhloufi et al., 2020). Indeed, diversity in groups can be based on variations in knowledge, skills, abilities, or expertise (*functional diversity or deep-level diversity*) or demographic characteristics such as gender, race, or culture (*demographic or surface-level diversity*). Results may differ in function of the type of diversity studied. It appears that *functional* diversity generally leads to positive effects on group performance (Hülsheger et al., 2009; Wang et al., 2019) whereas *demographic* diversity produces mixed findings (Bell et al., 2011; Reiter-Palmon et al., 2012; Schneid et al., 2016). It has been demonstrated that *demographic* diversity leads to negative performance, and is negatively related to innovation (Hülsheger et al., 2009). On the contrary, other findings showed that *demographic* diversity in teams (i.e., combination of gender, age, and national diversity) is moderately and positively related to team creativity in a real website design task (Curşeu, 2010). In another study examining the impact of both *demographic* and *functional* diversity on a task, participants repartitioned in 47 virtual dyads were asked to generate creative solutions to various human resource problems (Martins and Shalley, 2011). Results showed that nationality, age, and technical skills influenced creativity, while gender and race composition did not. Similarly, O’Reilly et al. (1998) found moderate positive effects for racial diversity on creativity while gender diversity had no effect or mixed effects (see also Pelled et al., 1999; Baer and Kaufman, 2008).

### Gender Diversity and Creative Performance

Specifically concerning gender diversity in groups and its effect on creativity, numerous empirical studies, meta-analyses and literature reviews have been conducted (Wood, 1987; Bowers et al., 2000; Webber and Donahue, 2001; Lee and Farh, 2004; Joshi and Roh, 2009; Kelemen et al., 2020), but no consistent effects of gender diversity on creativity were found. Studies demonstrated positive effects (Schruijer and Mostert, 1997; Curşeu et al., 2007; Díaz-García et al., 2013), negative effects (Cady and Valentine, 1999) or no effect (Herschel, 1994). For example, it was found in a study that mixed-gender groups produced more creative ideas (Schruijer and Mostert, 1997) and a higher number of alternatives (Curşeu et al., 2007) than same-gender groups. In a field study, Díaz-García et al. (2013) also showed that gender diversity within R&D teams fosters the discovery of novel solutions. On the contrary, other studies demonstrated that the higher gender diversity in teams, the lower the creativity (Cady and Valentine, 1999; Choi, 2007).

Confronted with these mixed findings, researchers suggested that gender diversity was not directly linked to group creativity, but instead depends on situational cues rendering gender more or less salient, such as stereotypical task content or saliency of gender characteristics within groups (van Knippenberg et al., 2004; Pearsall et al., 2008). According to the Categorization-Elaboration Model (van Knippenberg et al., 2004; van Knippenberg and van Ginkel, 2010), *demographic* characteristics such as gender provide a basis for categorizing oneself and others into different subgroups. As group members may belong to different social categories (Tajfel, 1982; Abrams et al., 1990; Turner et al., 1994; Turner, 2010; Turner and Reynolds, 2012), such as gender, they may self-categorized in “we” and “them,” and gender diversity may contribute to creating *faultlines* within groups (Lau and Murnighan, 1998; Pearsall et al., 2008; van Knippenberg and Hoever, 2018). In other words, when a difference on a characteristic is salient, it may serve to divide a group into subgroups, and subgroup formation may inhibit social interactions, reduce social cohesion (Mannix and Neale, 2005), and have deleterious impact on creativity (Kratzer et al., 2004). The negative effects of gender diversity may also be accentuated when the task is gender specific (Pearsall et al., 2008).

Based on these theoretical principles, it appears that *faultlines* negatively influence creativity in mixed-gender groups. Conversely, when gender categories were not salient in homogeneous groups, group performance improved (Jehn and Bezrukova, 2010), notably because each group member perceived that they belonged to the same gender category (van Knippenberg et al., 2011). In this perspective, it has been shown that all-men groups were more creative when working together on a tower-building task relative to mixed-gender groups and all-women groups (Gaggioli et al., 2019). Similarly, in a large field study involving 222 work group units, it was found that groups with a high proportion of female employees performed worse than mixed-gender groups, i.e., take greater time to complete a set of tax computations in groups (Wegge et al., 2008). As previously demonstrated in a meta-analysis (Wood, 1987), task content may influence the effectiveness of all-men and all-women groups. In the studies that favored all-men groups, the content of the task was more consistent with the stereotypical skills, interests, and abilities associated with men. Thus, the superiority of all-men groups in these studies may be partly explained by the stereotypical content of the tasks, consequently, stimulating performance of all-men groups and hindering performance of all-women groups (Wood, 1987; Bowers et al., 2000).

On the contrary, in a series of studies aiming to determine how collective intelligence develops in groups, and affects group performance, it has been demonstrated that groups composed of a higher proportion of women performed better than other groups, most notably on a synchronous electronic brainstorming task consisting of producing creative ideas (Woolley et al., 2010; Bear and Woolley, 2011; Engel et al., 2014; Malone and Bernstein, 2015; Woolley and Aggarwal, 2020). In these studies, groups composed of two to five members had to work together on tasks requiring different collaboration processes such as brainstorming, negotiation, mathematical reasoning, and moral-reasoning tasks (McGrath, 1984). Among other findings, it was noted that the proportion of women in groups was a significant predictor of group performance, and this effect was explained by the higher level of social sensitivity exhibited by women collaborating together (Hall and Schmid Mast, 2008; Williams and Polman, 2015). As recently noticed by Paulus and Kenworthy (2020), the mixed effects of gender composition in groups is of particular interest as studies on collective intelligence found that increasing the number of females in groups increases performance on a series of collaborative tasks, including idea generation. Beyond collective intelligence research, prior studies demonstrated that a higher female proportion within groups or teams lead to better group performance, even on a traditionally masculine (i.e., military) decision-making task (Hirschfeld et al., 2005). Similarly, it was found in a prior study that women experience the highest levels of collaboration in all-women groups, and collaboration declines as the proportion of men increases (Chatman and O’Reilly, 2004).

### Current Study and Hypotheses

It appears important to examine, in a large-scale study, whether the proportion of women/men in small groups may predict better creative performance. In our study, we will use a synchronous electronic brainstorming idea generation task to measure creativity. Although research compared all-men to all-women groups, or same-gender to mixed-gender groups, the Herschel (1994) study is the sole, to our knowledge, to have experimentally examined the effect of gender diversity in groups on idea generation by the means of a synchronous electronic brainstorming system, and no significant effects were found. However, it is worth noting that the number of groups in each condition was relatively small, and it was even dramatically small in all-women groups with only five groups. Recently, another small-scale study was conducted assigning participants to one of 20 online groups of five members to produce ideas using an asynchronous electronic brainstorming system (Coursey et al., 2020). It was observed that gender diversity is negatively related to the number of ideas (and novel ideas) generated in groups. In this study, the effect was observed even though individuals were not aware of the group members’ characteristics, including gender, but as suggested by the researchers they may have disclosed such information during group discussions.

Based on the literature review, two alternative hypotheses can be formulated depending on the situational cues provide by a gendered task and/or group composition, allowing one to categorize oneself and others into gender subgroups. Firstly, based on new insights provided by research on collective intelligence (Woolley et al., 2010; Woolley and Aggarwal, 2020) and collaboration in groups (Chatman and O’Reilly, 2004), we would predict negative effects of gender diversity in groups on creative performance, and specifically better performance in all-women groups than in all-men groups and mixed-gender groups (Hypothesis 1). Secondly, based on studies examining the effects of group and team diversity on performance (Wood, 1987; Bowers et al., 2000), we would expect that all-men groups would have better creative performance than all-women groups and mixed-gender groups, but only on a stereotypically masculine task (Hypothesis 2). In both hypotheses, it was difficult to predict, based on the disparate findings in the literature, which of the mixed-gender groups would lead to the lowest creative performance, groups with an equal number of men and women or “solo” groups with a single member of one gender among a majority of members of another gender.

The study was conducted on two cohorts of high school students as part of the large collaborative research project (ProFAN) launched in 2017 by the French Ministry of Education, and involving a large sample of vocational high schools with students from three different disciplines (business, health services, and electricity). The analyses focused on data from the initial baseline of the first and second cohorts, which have been acquired respectively in fall 2018 and 2019 from only the sample of business students.^1^

## Materials and Methods

### Participants

Participants were 2,261 business students (1,005 males and 1,256 females) from 64 vocational high schools in France and two cohorts (cohort 2018 = 975 and cohort 2019 = 1,286). The great majority of students were 16 or 17 years old (53.6 and 28.7%, respectively), 8.1% were 15 years old, 6.3% were 18 years old, and the remaining students were under 14 years old (0.4%) or more than 19 years old (2.4%). In total, all participants were divided into 711 groups, 583 three-member groups (*n* = 1,749) and 128 four-member groups (*n* = 512). Each group was composed of students in the same classroom, and therefore enrolled in the same vocational high school. Although participants were randomly distributed in groups, for statistical analyses groups were then categorized (a posteriori) into five categories where the proportion of men and women varied: (1) all-men groups (*n* = 164, 48 three-member groups and 5 four-member groups), (2) “solo” groups of one-woman and two- or three-men (*n* = 681, 199 three-member groups and 21 four-member groups), (3) groups of two men and two women (*n* = 184, 46 four-member groups), (4) “solo” groups of one-man and of two- or three-women (*n* = 910, 242 three-member groups and 46 four-member groups), and (5) all-women groups (*n* = 322, 94 three-member groups and 10 four-member groups).

### Procedure

Students completed the study in a high school computer room during regular school hours under the supervision of one of their teachers. Each student was individually seated in front of a computer for both the questionnaire and the brainstorming task.

**FIGURE 1:**
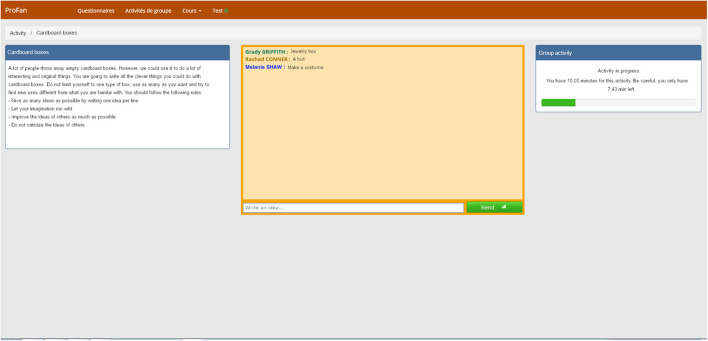
Screenshot of the synchronous electronic brainstorming system.

First, students completed a web-questionnaire composed of a series of scales, including the creative cognition scale to control individual differences on creativity. Second, about one month after filling in the questionnaire, students were seated in the same computer room in half-classrooms, and completed a series of tasks assessing their collaboration skills. They logged in to a digital toolbox specifically built for the project and containing four tasks, including an idea-generation task performed using a synchronous electronic brainstorming system similar to that used by Woolley et al. (2010). Once connected to the system,^2^ each group was composed of students in the same classroom (and therefore from the same vocational high school). Of the four tasks to be completed, the first was an idea-generation task performed by the means of an electronic brainstorming system, which is the only task discussed in the current study. Half of the high schools had been randomly assigned to the cardboard box condition (neutral task), the other half were assigned to a metal box condition (gendered task).^3^ Students were instructed to generate as many ideas as possible on how the object could be recycled for new purposes, following the Osborn (1957) four brainstorming rules: focus on quantity, withhold criticism, welcome unusual ideas, and combine and improve ideas. In order to communicate with each other, students shared their ideas by using a chat window. Each idea was displayed on the screen alongside the student’s name who produced it. Also, each participant was aware of the gender of their group-members during the task by their surname and first name which appeared in the chat (see Figure 1).

After performing the synchronous electronic brainstorming task, students had to perform other collaborative tasks implemented into the digital toolbox involving sharing information, collective decision making, and coordination processes. As these tasks did not measure creativity, they were not examined in the present study.

### Measures

#### Creative Performance

The three main criteria of divergent thinking (Guilford, 1950) were used as indicators of creative performance: the number of non-redundant ideas produced (fluency), the number of semantic categories of ideas mobilized (flexibility), and the novelty of ideas (originality).

##### Fluency

All the non-redundant ideas produced by each participant were counted by four coders previously trained on the coding of ideas (*N*_*ideas*_ = 1,592). Higher scores represent greater fluency. Overall, individual participants generated an average of 6.89 ideas (*SD* = 5.86) and groups generated an average of 21.91 ideas (*SD* = 14.16) during the 10 min of the electronic brainstorming session.

##### Flexibility

A three-step procedure was used to code flexibility. In a first step, a group of three students in psychology (two women and one man) were instructed to create semantic categories from a list of 200 ideas randomly extracted from the 1,592 non-redundant ideas on both tasks (cardboard and metal box). They also had to assign each idea to one category. Ten semantic categories were then created: (1) Furniture and household items, (2) Arts, (3) Office supplies, (4) Flora and fauna, (5) Mechanics, DIY and tools, (6) Clothing, jewelry and accessories, (7) Games and sports, (8) Containers, (9) Buildings, and (10) Transportation. In a second step, 20 students blind to the hypotheses had to categorize the same sample of 200 unique ideas into the categories previously created. Using an online card sorting system,^4^ they had to drag-and-drop each of these ideas into one of the ten categories. Results showed that 84% of the ideas were arranged in the same categories as those chosen by the three coders at the first step, demonstrating the validity of the original categories created by the first coders. In a third step, four coders had to assign the 1,592 ideas to one of the ten categories. A Fleiss Kappa was calculated and showed an acceptable inter-rater agreement between the four coders (*k* = 0.78). Thus, a flexibility score ranging from 0 (no idea produced and therefore no semantic category explored) to 10 (all semantic categories) was computed for each participant. Higher scores represent greater flexibility.

##### Originality

All ideas were scored on an originality criterium on a Likert scale, ranging from 1 (*very common idea*) to 5 (*very original idea*). An individual originality score based on the sum of originality scores of ideas produced by each participant was computed by a subjective assessment of each coder who rated all the ideas. Four coders were trained to rate the originality and subsequently rated all non-redundant ideas (*N* = 1,592) for their originality. As an acceptable intraclass coefficient between all coders’ ratings was obtained (*ICC* = 0.72), an average of the scores of the four coders was computed. Higher scores represent greater originality in the ideas produced by an individual.

#### Control Variable

In order to control individual differences on creativity, and because of its potential relation to creative performance, participants had to complete a creative cognition scale (*Use of Creative Cognition Scale*, Rogaten and Moneta, 2015a) on a Likert scale ranging from 1 (*never*) to 6 (*always*). Sample items were: “I find effective solutions by combining multiple ideas,” “While working on something, I try to generate as many ideas as possible.” Participants completed this scale about one month before the brainstorming task. A principal component analysis revealed one factor explaining 62.65% of the variance. Moreover, confirmatory factor analysis supports the one-factor structure of the original scale although “the model does not fit strictly” as in the original scale (Rogaten and Moneta, 2015a, p. 308), χ*^2^* = 184, df = 5, *p* < 0.001 (CFI = 0.96; TLI = 0.92; RMSEA = 0.13). A creative cognition score was computed for each participant by averaging scores of five items (Cronbach’s alpha = 0.85). Higher scores indicate greater creative cognition.

## Results

Data were modeled using multivariate generalized linear mixed models (mGLMMs) with distinct random intercepts because this analytic strategy offers several advantages. First, it is particularly suitable when conditional distributions of outcomes given covariates and random effects are non-normal (McCullagh and Nelder, 1989). Second, it takes into consideration the associations between the different measures of creative performance (i.e., Fluency, Flexibility, and Originality) whilst examining the impact of predictors (Gender Diversity, Task) and covariates (Cohort, Creative Cognition), offering a better control over Type I error and more consistent and accurate estimates and inferences. Finally, it allows for modeling clustered outcomes by incorporating the correlations between the outcomes through correlated random intercepts that are used to model individual and group specific effects.

A mGLMM was specified for estimating the impact of Gender Diversity both on Fluency, Flexibility and Originality accounting for Task effect (1 for the gendered task, 0 for the neutral task) and covariates (Cohort and Creative Cognition). Since the Gender Diversity predictor had five levels, it was recoded into a series of four dummy contrast variables with level 5 (all-women groups) chosen as the reference level: GD1 (1 if an individual belonged to an all-men group, 0 otherwise), GD2 (1 if an individual belonged to a “solo” group with one-woman, 0 otherwise), GD3 (1 if an individual belonged to groups of two men and two women, 0 otherwise) and GD4 (1 if an individual belonged to a “solo” group with one-man, 0 otherwise). In all, seven fixed effect predictors were included in the model: one continuous predictor (Creative Cognition), six dichotomous variables (Cohort, Task, GD1, GD2, GD3, and GD4) plus a fixed intercept. In addition, since observations were nested within individual (three creativity scores for each subject) but also clustered according to type of task, the model incorporated two random intercepts for every outcome. The first random intercept represented the individual-specific effect and the second random intercept represented the group-specific effect. The correlation structure among the outcomes was therefore assumed to be captured by the correlations between the three individual-specific random intercepts and by the correlations between the three group-specific random intercepts.

After determining relevant conditional distributions of data for each outcome (Negative-binomial distribution for Flexibility and Fluency, and Gamma distribution for Originality), our aim was to find a parsimonious model with the highest possible predictive performance. We conducted model building using a backward model selection before final parameter estimation and inference (see online supplementary analyses of our OSF project at https://osf.io/cz9h2/), and we used the AIC for selecting the “best” model. The candidate model with the lowest AIC value being considered the model that best fitted the data. The mGLMMs were fitted using the “fitmv” function of the spaMM package in R (Rousset and Ferdy, 2014). Model parameters were estimated by restricted maximum likelihood estimation (REML) based on the Laplace approximation. The comparison using AIC of all our tested mGLMMs led us to consider the model with Task, Gender Diversity and Creative Cognition as predictor variables as the most parsimonious model for the given data. Since the log of the expected count is modeled as a function of the predictor variables, the formula for the predicted (conditional) mean value of each creative performance measure is: Creative Performance = exp^(β0^^+^^β1^^∗^^T^^+^^β2^^∗^^CC^^+^^β3^^∗^^*GD1*^^+^^β4^^∗^^*GD2*^^+^^β5^^∗^^*GD3*^^+^^β6^^∗^^*GD4*)^ with T is the task (gendered vs neutral), CC is the Creative Cognition, and GD is Gender Diversity (GD 1: all-men groups vs all-women groups; GD 2: “solo” groups with only one-woman vs all-women groups; GD 3: groups of two men and two women vs all-women groups; GD 4: “solo” groups with only one-man vs all-women groups).

Table 1 provides fixed and random effects estimates for this model. Regression coefficient estimates (Bs) are unstandardized and are on the logarithmic scale. They are followed by their estimated conditional standard errors (cond SEs), the Wald tests (t = $\frac{E\,s\,t\,i\,m\,a\,t\,e}{S\,E})$which are normally distributed and their corresponding p-values. For each estimate, exp(B) is referred to as the Incidence Rate Ratio (IRR). Table 1 also shows estimates of the variances of the individual- and group-specific random intercepts.

**TABLE 1:** Summary of the “best” multivariate generalized linear mixed model predicting participants’ creative performance for the three measures: fluency, flexibility, and originality (*N* = 2,261).

**Fluency**							
**Effect**	**B**	**95% CI for B**		**SE B**	**t**	**exp(B)**	**pseudo-r^2^**
		***LL***	***UL***				
Fixed effects							1.96
Intercept	1.587***	1.45	1.85	0.088	17.95	4.889	
Task	0.116*	0.024	0.207	0.053	2.200	1.123	
Creative	0.012***	0.004	0.017	0.003	3.218	1.012	
cognition							
Gender diversity							
GD1	−0.226*	−0.420	−0.011	0.118	−1.919	0.798	
GD2	−0.320***	−0.427	−0.141	0.083	−3.871	0.726	
GD3	−0.185	−0.402	0.114	0.119	−1.556	0.831	
GD4	−0.159*	−0.293	−0.119	0.079	−2.018	0.853	
Random effects							
Individual variance	0.428						
Group variance	0.285						

**Flexibility**							
**Effect**	**B**	**95% CI for B**		**SE B**	**t**	**exp(B)**	**pseudo- r^2^**
		***LL***	***UL***				

Fixed effects							0.80
Intercept	1.172***	1.10	1.38	0.068	17.220	3.228	
Task	0.022	−0.032	0.094	0.039	0.570	1.022	
Creative	0.007*	0.002	0.012	0.003	2.082	1.007	
cognition							
Gender diversity							
GD1	−0.097	−0.254	0.028	0.088	−1.102	0.908	
GD2	−0.140**	−0.230	−0.034	0.061	−2.277	0.869	
GD3	−0.058	−0.211	0.070	0.088	−0.666	0.944	
GD4	−0.063	−0.161	0.026	−0.059	−1.079	0.939	
Random effects							
Individual variance	0.185						
Group variance	0.121						

**Originality**							
**Effect**	**B**	**95% CI for B**		**SE B**	**t**	**exp(B)**	**pseudo- r^2^**
		***LL***	***UL***				

Fixed effects							1.96
Intercept	1.874***	1.77	2.16	0.115	16.295	6.514	
Task	0.172**	0.101	0.270	0.067	2.550	1.188	
Creative	0.013**	0.005	0.019	0.005	2.614	1.013	
cognition							
Gender diversity							
GD1	−0.163	−0.336	0.042	0.151	−1.080	0.850	
GD2	−0.276**	−0.361	−0.096	0.106	−2.604	0.759	
GD3	−0.137	−0.355	0.231	0.152	−0.905	0.872	
GD4	−0.134	−0.261	−0.006	0.102	−1.314	0.875	
Random effects							
Individual variance	0.675						
Group variance	0.417						

*Dummy variables with all-women groups chosen as the reference: GD1 (all-men groups vs all-women groups), GD2 (“solo” groups with one-woman vs all-women groups), GD3 (groups of two men and two women vs all-women groups) and GD4 (“solo” groups with one-man vs all-women groups).*

*The CI values are obtained by estimating each of the three GLMMs separately while corresponding parameters are simultaneously estimated in the mGLMM.*

*p < 0.05, **p < 0.01, ***p < 0.001.

**FIGURE 2:**
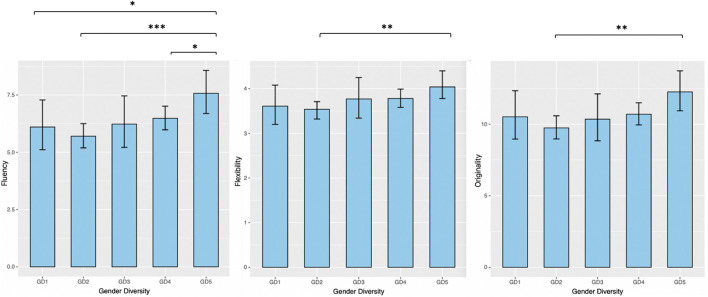
Estimated marginal means (predicted values) of creative performance scores for each level of Gender Diversity. GD1: all-men groups; GD2: “solo” groups of one-woman and two- or three-men; GD3: groups of two men and two women; GD4: “solo” groups of one-man and of two- or three-women; GD5: all-women groups. Intervals are 95% confidence intervals for the predicted values adjusted for task and creative cognition. Predictions are on the population-level and do not account for the random effect variances. *p < 0.05, **p < 0.01, ***p < 0.001.

As shown in Table 1, firstly, it appears that performance on the gendered task compared to the neutral task, while holding the other predictor variables constant in the model, is expected to be 1.123 times higher for Fluency. A similar result is observed for Originality but not for Flexibility, suggesting that more ideas (and original ideas) are found for the metal box task than for the cardboard box task. Secondly, an effect of creative cognition on creative performance is observed, and shows that when the participant’s creative cognition increases by one point, the Fluency score would be expected to increase by a factor 1.012 while holding all other predictor variables constant. In other words, the higher creative cognition, the higher creative performance. Similar results are found for both Flexibility and Originality, revealing a positive impact of the control variable on the three measures of creative performance. Finally, in comparison to all-women groups, and holding constant the other predictor variables in the model, the Fluency score is expected to be 0.726 times lower in “solo” groups with one-woman (GD 2), and similar results are observed for the two other measures. The same results are observed for GD 1 and GD 4, suggesting that all-women groups have a better creative performance on fluency, but not on flexibility and originality, than all-men groups and “solo” groups. However, this effect is not observed for groups composed of two men and two women which are not significantly different from the estimates for the reference group. For the two other measures of creative performance (Flexibility and Originality), only the difference between all-women groups and “solo” groups with one-woman is significant. Thus, all women groups produced a wider range of ideas and more creative ideas than “solo” groups where only one woman was present in the group (see Figure 2).

## Discussion

The aim of the study was to examine the effects of gender diversity in groups on creative performance by using a synchronous electronic brainstorming task. We observed these effects on three measures of creative performance (fluency, flexibility and originality), considering gender diversity, stereotypical nature of the task (gendered vs neutral), and the participants’ individual differences on creative cognition as predictors.

To begin with, results reveal that creative cognition, an individual differences variable on creativity, influences creative performance. Although creative cognition was only used in the present study as a short self-report measure for controlling individual differences on creativity before the idea-generation task, a positive effect was observed on the three measures of creative performance. Although this result is not at the core of the present research, it provides a first empirical demonstration about the predictive validity of creative cognition in educational settings, beyond the initial work of Rogaten and Moneta (2015a). The present study only highlighted a positive effect of the self-reported creative cognition measure on idea-generation task. Future studies should enrich these results examining the predictive validity of the creative cognition scale on creative tasks involving different cognitive processes (e.g., convergent thinking). In addition, it would be fruitful to use other measures of individual creativity, such as creative self-efficacy scale (Karwowski et al., 2018) or creative abilities (Rogaten and Moneta, 2015b).

Related to our main research question in this study concerning the influence of gender diversity in groups on creativity, two alternative hypotheses can be formulated. The first hypothesis is based on collective intelligence research (Woolley et al., 2010; Woolley and Aggarwal, 2020), in which better creative performance should be observed in groups with a higher proportion of women than in other groups. Results revealed that this hypothesis is partially supported. Indeed, all-women groups have better creative performance, particularly on fluency, than all-men groups and “solo” groups composed of only one woman/man among a majority of men/women. However, there is no difference between all-women groups and mixed-gender groups composed of an equal number of men and women. These findings suggest that gender diversity has a negative impact on creative performance, but only in “solo” groups where the gender differences are more likely rendered salient by the presence of a distinctive member. Indeed, a single person of one gender among a majority of people of the other gender may provide situational cues rendering gender more salient, and leading to the creation of a division in groups. On the contrary, when group members have the possibility to form sub-groups (here, dyads) composed of people of same gender, no differences are observed relative to all-women groups. Although speculative, it is reasonable to consider that in these groups, individuals generate more creative ideas because of a greater identification with their sub-group, and allocated no attention to the other sub-group. Everything seems to happen as if the two same-gender subgroups functioned independently to each other, and the formation of sub-groups/dyads may have prevented the creation of *faultlines* in the equilibrated gender groups. Of course, this interpretation remains speculative as we did not measure participants’ perception of the group dynamics. However, according to the self-categorization theory (Abrams et al., 1990; Turner et al., 1994; Turner, 2010; Turner and Reynolds, 2012), gender cues may be used to distinguish between individuals assigning them implicitly in social groups. Such an interpretation refers to the socio-cognitive conception of the social groups where group members do not need to interact to feel themselves members of the group, but it is enough that they mentally represent themselves their own group in comparison to another group.

The better creative performance observed in groups with a higher proportion of women in collective intelligence research, has previously been explained by more balanced “speaking turns” during the idea-generation task. However, such an interpretation is unlikely to be at work in the present study, because one of the main advantages of electronic brainstorming is that it allows the participants to give their ideas at any moment during the session, preventing “production blocking” processes which might occur in face-to-face groups (Gallupe et al., 1991; Nijstad et al., 2003). Nevertheless, our results are partly consistent with studies on collective intelligence (at least for the brainstorming task) since groups with the highest proportion of women outperform the other groups, at least on fluency, except for groups with an equal number of men and women in two distinctive gender dyads. Furthermore, our results go beyond collective intelligence research in demonstrating that all-women groups have better creative performance than “solo” groups, and particularly groups composed of one woman among a majority of men.

Taken together, these results suggest that social categorization would be more salient in “solo” groups with only one woman or man, leading to a detrimental sub-division of the group on creativity (Lau and Murnighan, 1998; Kratzer et al., 2004). Indeed, “solo” status emphasizes gender differences in the group since attention is focused on the “solo member,” making his/her social category and differences in groups more salient (Twuyver and Knippenberg, 1999; Thompson and Sekaquaptewa, 2002), and leading to the emergence of *faultlines* in “solo” groups. Some studies revealed that men and women experienced “solo” status differently (Thompson and Sekaquaptewa, 2002; Chatman et al., 2008; Viallon and Martinot, 2009) which may explain why differences between all-women groups and “solo woman” groups were found on the three measures of creative performance. As “solo” status affects women more negatively than men, a sole woman in a group of men tends to be more anxious of being perceived negatively by other group members (Saenz and Lord, 1989), and less engaged in the task than a sole man in a group of women (Sekaquaptewa and Thompson, 2003). In our study, the perception to be “solo” in a group, may have also contributed to create division or *faultlines* within the group. To our knowledge, no study has questioned effects of “solo” status on idea generation. Thus, we can suppose that “solo” status more negatively affects creative performance in “solo woman” groups than “solo man” groups. Investigating this question would be a new promising avenue for future studies. The results about detrimental effects in “solo” groups on creative performance could also be explained by the model of “representational gaps” in teams (Weingart et al., 2005; Cronin and Weingart, 2007). According to this model, the gaps cause team members to perceive the group’s task differently, and may lead to lower creativity in cross-functional teams which develop new products (Weingart et al., 2008). It is possible to extend this reasoning to the present study where “representational gaps” may have emerged in “solo” groups when gender characteristics are salient. Further studies would need to examine possible “representational gaps” based on gender diversity in groups and differences in performance on stereotypically gendered tasks. To our knowledge, as creative performance measured by the generation of ideas in a brainstorming task has never been explored in “solo” groups, this large-scale study appears to be a good starting point for further investigating the underlying mechanisms in studies at a smaller scale.

The second alternative hypothesis argued that all-men groups should have better creative performance than other groups, and particularly mixed-gender groups, but only on the stereotypically masculine task which consisted of producing as many ideas as possible about the uses of a metal box (Wood, 1987; Bowers et al., 2000). This prediction did not receive empirical support in the present study. Instead, results revealed only an effect of the task on fluency and originality, for all groups. For unclear reasons, participants produced more ideas and more original ideas when they completed the gendered task (metal box) than the neutral task (cardboard box). Although the two tasks are structurally similar (i.e., they are both boxes and can serve as containers), it is possible that the pretested masculine task in the present study is not sufficiently stereotyped to be used as situational cues to subdivide mixed-gender groups, and consequently, to negatively impact women’s creative performance. Although the pretest of the gender stereotypically associated with the tasks revealed that the metal box was perceived as a more masculine task than the cardboard box, the differences between the tasks may not be great enough to produce a strong effect. This is one of the limitations of the present study, and it would be relevant in future studies to vary the type of gendered tasks, and to examine the effects on creative performance in “solo” groups relative to homogeneous groups.

Finally, it is reasonable to suppose that our findings, revealing better creative performance of all-women groups in comparison to “solo” groups and all-men groups, can be explained by individual differences between men and women on divergent thinking rather than gender group diversity *per se*. Indeed, the results are consistent, at least in part, with studies identifying a superiority of women in verbal creativity tasks (Abraham, 2016), and can be explained by a generally higher verbal fluency among women (Weiss et al., 2006). Although some literature on gender and creativity reports differences between men and women on divergent thinking tasks where women score higher, these differences are generally counter-balanced by a number studies demonstrating opposite findings (Baer and Kaufman, 2008). As pointed out by Abraham (2016), approximately half of the investigations reported no significant differences between male and female participants on creativity, whereas the other half were characterized by mixed findings suggesting, on average, superior creative abilities among female participants. As the idea-generation task used in our study is a divergent thinking task where performance in groups is the sum of the individual contributions, creative performance should increase as the proportion of women in the groups increases. This is not what we observed because, first, no difference was found between all-women groups and mixed-gender groups composed of two men and two women, and second, the main difference observed is between all-women groups and “solo” groups composed of only one person of one gender, and especially “solo” groups composed of only one woman.

Although this research examines divergent thinking on a large sample, there are limitations to underline. As with much research on team performance, our study involved a student sample. Conducting this type of research in other settings with different populations would be valuable in demonstrating the generality and applicability of our findings. However, when researchers have examined group creativity in organizations, the findings have been similar to those of student samples (Paulus et al., 1995; Paulus and van der Zee, 2015). Moreover, we have no empirical studies showing differences between work teams and student groups concerning the activation of gender *fautlines* and the salience of gender differences. Because the issue of gender diversity in work teams is of major interest, future studies should be conducted with older populations. Another limitation is that the effect of group diversity was only tested for divergent thinking. Moreover, the measurement of the different indicators of divergent thinking is subject to many debates (Runco et al., 1987; Silvia et al., 2008), in particular with regard to the strong relationship between the three classic indicators and the confounding effect of fluency in other divergent thinking scores (Forthmann et al., 2020). Also, to increase the validity of the study, further research should be conducted using other indicators of creativity such as integrative thinking tasks, insight tasks, or product-based assessment. Finally, we examined groups in the early stages of interaction where *faultline* activation and idea generation occurred during a short period of time. During a divergent thinking task, it has been recognized that the number of ideas decreases over time (Beaty and Silvia, 2012; Wang et al., 2019). As time is a crucial variable in idea generation (Said-Metwaly et al., 2020), further studies should examine more thoroughly short versus long time (electronic) brainstorming sessions on creative performance. Indeed, the influence of surface-level characteristics such as gender may decline over time as team members build relationships based on non-demographic characteristics such as personality or values (Jehn et al., 1999; Price et al., 2002). Future research is needed to generalize the data and further explore the effects of time in brainstorming session on creative performance.

In closing, this large-scale study highlighted the effects of gender-group composition on the generation of creative ideas. In particular, it revealed a negative effect of “solo” status in mixed-gender groups on idea generation and, thus, paves the way for future studies to further explore these results.

**Members of the ProFAN Consortium:** Batruch Anatolia, Butera Fabrizio, Rudmann Ocyna (Social Psychology Laboratory, University of Lausanne, Lausanne, Switzerland), Desrichard Olivier, Mella-Barraco Nathalie, Ofosu Nana (Faculty of Psychology and Educational Sciences, University of Geneva, Geneva, Switzerland), Visintin Emilio Paolo (Humanities Department, University of Ferrara, Ferrara, Italy), Brown Genavee (Pact Lab, Nothumbria University, Newcastle upon Tyne, United Kingdom), Bressan Marco, Poletti Céline, Régner Isabelle, Vives Eva (Cognitive Psychology Laboratory, Aix-Marseille Univ, Marseille, France), Bressoux Pascal, De Place Anne-Laure, Pansu Pascal, Riant Mathilde, Sanrey Camille (LaRAC, University of Grenoble Alpes, Grenoble, France), Cherbonnier Anthony, Goron Luc, Hemon Brivael, Jamet Eric, Michinov Estelle, Michinov Nicolas, Peter Laurine (Laboratory of Psychology: Cognition, Behavior, Communication, Univ Rennes, Rennes, France), Darnon Céline, Demolliens Marie, Huguet Pascal, Robert Anais, Stanczak Arnaud (Social and Cognitive Psychology Laboratory, University of Clermont Auvergne, Clermont-Ferrand, France), Bouet Marinette, Cepeda Carlos, Ducros Théo, Martinez Ruben, Mazenod Vincent, Petitcollot Benoit, Toumani Farouk, Vilmin Simon (LIMOS, University of Clermont Auvergne, Clermont-Ferrand, France).

## Data Availability Statement

The datasets for this study can be found on the OSF page of the project available at: https://osf.io/cz9h2/.

## Ethics Statement

Ethical review and approval was not required for the study on human participants in accordance with the local legislation and institutional requirements. Written informed consent to participate in this study was provided by the participants’ legal guardian/next of kin.

## Author Contributions

LP and NM drafted and edited the manuscript. JJ analyzed the data. MB, GB, EM, and EJ critically revised the manuscript. AC contributed to the ergonomics design of the digital toolbox and performed the user tests. All authors listed have made a substantial, direct and intellectual contribution to the study, and approved it for publication.

## Conflict of Interest

The authors declare that the research was conducted in the absence of any commercial or financial relationships that could be construed as a potential conflict of interest.

## Publisher’s Note

All claims expressed in this article are solely those of the authors and do not necessarily represent those of their affiliated organizations, or those of the publisher, the editors and the reviewers. Any product that may be evaluated in this article, or claim that may be made by its manufacturer, is not guaranteed or endorsed by the publisher.

## Footnotes

1 Only the sample of business students was used in the present study because it was the most equilibrated on gender. The two others samples, composed of students in health services and electricity, were not equilibrated on gender composition, the former had a large majority of females (95%) and the latter a large majority of males (98%).

2 By default, the electronic brainstorming system automatically randomized participants in three-member groups, and when the count was not exact in half-classrooms, four-member groups were built.

3 In order to identify the gender stereotypically associated to the tasks, a pre-test was carried out with 62 high school students (29 females and 33 males) aged from 15 to 19 years old (*M*_*age*_ = 16.13 and *SD* = 1.09), and not involved in the large-scale experiment. A list composed of ten items (paper clip, brick, cardboard box, newspaper, spoon, sponge, shelf, metal box, door and pen) was presented; participants had to identify on an eight-centimeter continuum scale from 0 (*totally a masculine item*) to 8 (*totally a feminine item*), who, men or women, would be the most able to find alternative uses for each of these objects. A *t*-test for paired samples reveals that the metal box is perceived as more masculine (*M* = 3.12 and *SD* = 1.58) than the cardboard box (*M* = 4.14 and *SD* = 1.7), *t*(61) = 3.47, *p* < 0.01. The latter can be considered as a neutral task because of the mean value is very close to the theoretical mean.

4
https://www.optimalworkshop.com
